# Disparities in telehealth use: How should the supportive care community respond?

**DOI:** 10.1007/s00520-021-06629-4

**Published:** 2021-10-19

**Authors:** Niharika Dixit, Ysabella Van Sebille, Gregory B. Crawford, Pamela K. Ginex, Paz Fernandez Ortega, Raymond J. Chan

**Affiliations:** 1 Department of Medicine, Division of Hematology & Oncology, University of California, San Francisco at Zuckerberg San Francisco General Hospital, San Francisco, CA USA; 2 University of South Australia Online, Adelaide, Australia; 3 Northern Adelaide Local Health Network, Adelaide, Australia; 4 Discipline of Medicine, University of Adelaide, Adelaide, Australia; 5 Evidence-Based Practice and Inquiry, Oncology Nursing Society, Pittsburgh, PA USA; 6 Institut Català d’Oncologia, Barcelona, Spain; 7 College of Nursing and Health Sciences, Caring Futures Institute, Flinders University, Adelaide, Australia

**Keywords:** Telehealth, Health equity, Cancer care, Barriers in access

## Abstract

Telehealth use has increased in the setting of the COVID-19 pandemic. However, there are disparities in telehealth use based on age, income, race/ethnicity, low health, digital literacy, and limited English proficiency. There are multilevel barriers to telehealth use at the patient, health systems, telehealth portal, and policy levels. To ensure equity in telehealth services and to leverage these services to maximize the reach of health care services, concerted efforts are needed to design telehealth tools and workflows. It should include reimbursement for staff training, patient education, and technical support needed for telehealth use. Furthermore, ongoing monitoring and responsive modifications in the use of telehealth services are needed to promote telehealth equity.

## Introduction

In March 2020, the World Health Organization (WHO) declared SARS-CoV-2 (COVID-19) a pandemic [1]. Many local, state, and national governments instituted guidelines for physical distancing [2, 3], and health systems expanded telehealth quickly, and telehealth visits increased across the USA and worldwide, including for supportive and survivorship care in cancer [4, 5]. The policy changes in many countries with coverage of the telehealth visit at the same level as an in-person visit facilitated this transformation. Center for Medicare and Medicaid Services (CMS) defines telehealth as the exchange of medical information from one site to another via electronic communication [6]. It includes telecommunication technologies to support distant clinical health care, patient and professional education, public health, and health administration [6]. For this commentary, we focus on patient-facing telehealth, including audio or video clinical encounters, patient access to their medical records and their medical team, and patient education interventions delivered by telehealth approaches.

Telehealth is expected to remain an essential tool for cancer care beyond the pandemic, including in easing the backlog caused by the pandemic, and notably it can play a critical role in supportive care in cancer. Moreover, global surveillance reports suggest a trend toward increased cancer survival and chronicity, [7] increasing the demand for supportive and survivorship care services. Additionally, the increased availability and use of oral antineoplastics have reduced the need for in-person visits but increased the need for long-term monitoring for toxicities and medication adherence [8]. Thus, telehealth presents a unique opportunity to support optimal patient-centered care integrating cancer treatment with patient-directed supportive and palliative care. Further, telehealth can extend services that may not be available locally and is convenient for patients; for example, telehealth delivered genetic counseling, psycho-oncology, palliative care, nutritional services, and survivorship follow-up services not requiring physical examination [9].

However, it is well known that cancer disparities persist through the continuum of care, including supportive and survivorship care [10–12]. Patients who have low income [4], have limited English proficiency (LEP) [13], are older adults [14], have low health literacy, and receive care in public hospitals or rural hospital settings have limited access to telemedicine [4, 13–15]. In addition, they are also more likely to receive suboptimal supportive care interventions and experience a higher symptom burden during and after cancer treatments [16–19]. If concerted efforts are not made to address equity in telehealth, it is more likely to exacerbate pre-existing disparities in supportive and survivorship care. This commentary describes the multilevel barriers to telehealth and proposes steps to address these inequities in telehealth.

## Barriers to telehealth can be classified into four different levels requiring multilevel approaches to address disparities

1. Patient level: Older adults who require care of chronic diseases in addition to cancer are likely to have lower access to digital health tools, including lower access to internet, smartphone ownership, and digital health access tools [14]. Together, this reduces access to telehealth video visits [4, 14]. Furthermore, it is exacerbated by hearing deficits and complicated by late effects of cancer treatment, such as chemotherapy-related cognitive impairment and peripheral neuropathy. Similarly, low-income individuals have lower rates of smartphone ownership and access to the internet [13] and lower rates of engagement with telehealth [13]. Patients with LEP may not gain the level of care required due to misunderstanding care delivered via telehealth and may even be excluded from telehealth video visits if interpretation services are not included in the systems [13]. For example, 33% of rural Americans lack access to internet that can support telehealth video visits while also experiencing higher chronic disease burden and lower access to health services [20]. Nouri et al. reported lower telehealth use in Black/African American and Latinx patients in an urban safety net, highlighting the role of entrenched systemic racism in health care[21].
2. Health system level: Very few health care systems had robust telehealth use before the pandemic except tele-dermatology [22] and organizations like Kaiser Permanente [23, 24] that had implemented telehealth visits before the pandemic. At the health system level, the clinic workflows designed for in-person visits need optimization for telehealth to ensure that critical care is not missed, and team-based care is seamlessly integrated into a telehealth model.
3. Telehealth systems: Current telehealth digital systems are complex and not designed for accessibility for older patients and for patients who may have limited digital literacy [25]. They are also not optimized for smartphone use, which is more likely to be used by low-income individuals and crucial for internet access in low- and middle-income countries. Most telehealth systems have limited functionality for medical interpreters’ use, thus creating barriers for individuals who have LEP.
4. Policy level: At the policy level, the most significant barrier to telehealth is the reimbursement model that prioritizes in-person visits [26]. In addition, lower reimbursement for audio-only versus video visits is likely to penalize institutions that provide care for medically underserved patients and is likely to discourage telehealth use. Such limitations discourage institutions that deliver audio-only encounters to provide telehealth services and serve rural and low-income patients.

## Potential solutions

The increase in telehealth entails great opportunities to increase patients’ access to cancer professionals and to streamline the workflow of health care providers during and beyond the COVID-19 period. Furthermore, telehealth holds tremendous potential for the transforming the follow-up care, with a reduced burden for in-person visits. However, important concerns relating to associated regulatory frameworks, digital poverty and exclusion, and the respect of patients’ preferences need to be addressed concomitantly to its deployment. Here, we present a clear multilevel strategy and development of best practices required to address these barriers (Table 1). First, at the patient level, we recommend a comprehensive assessment for patient-level barriers, including readiness to use telehealth, access to broadband, disabilities that limit telehealth use, and limited digital literacy. Telehealth access can be improved through interventions such as patient-level training, voice-activated commands, simpler designs, engaging informal caregivers, and finally improving interpreters’ access to the telehealth portal. Second, at the health system level, it is crucial to design clinical workflows with a health equity lens to *not* exacerbate existing disparities but to increase access to care. To be genuinely successful, patient education and training for digital and telehealth tools must be built within clinical workflows to address disparities in access. While this training is often time-consuming and resource-intensive, it is an investment in excellent cancer care that is likely to increase patient engagement. One potential option is to leverage lay health workers and navigators for this type of training. Third, telehealth systems were primarily designed for business community and have not been optimized for team-based care including interpreters, volunteers, and administrative personnel. These systems should address these barriers, informed by patient experience, and incorporate feedback from end-users, including both clinicians and patients on an ongoing basis. Finally, telehealth can be improved at the policy level by continuing reimbursement for telehealth; setting requirements for telehealth systems including ease of access, privacy, reimbursement for time, and resources for patient training; and increasing support for access to broadband and telehealth devices for low-income individuals. To gain additional benefits from digital technologies, greater personalization, monitoring, and engagement of patients with digital solutions must be integrated into services.

**Table 1:** Barriers to telehealth and potential solutions to promote health equity

Barriers	Potential solutions	Suggested outcome measures
*Patient level*		
Inexperience with telehealth Low digital literacy Access to devices Access to broadband Limited English Proficiency	Assess readiness to use telehealth Provide training and technical support Ensure access to devices Ensure access to broadband Availability of interpreters for telehealth encounters Engagement of informal caregivers	Uptake of telehealth use and ongoing use at patient level Access to telehealth Patient satisfaction with visits
*Health system level*		
Lack of trained personnel Lack of optimized workflow	Training clinical staff Creating workflows optimized for telehealth use, including multidisciplinary team-based care Training and technical support for patients	Staff engagement in telehealth Telehealth visits volumes and time and quality measures for care
*Telehealth tools*		
The complexity of telehealth tools Poorly designed for accessibility	Simple design and interface informed by patient and provider feedback Tools designed for team-based care Easy to use applications designed for smartphone use	Patient and provider reported measures of usability
*Policy level*		
Reimbursement model prioritizing in-person visits Lower reimbursement of audio only visits No accessibility standards required for telehealth tools	Parity for telehealth visits including audio visits Reimbursement for patient telehealth education initiatives Mandating accessibility in telehealth tools	Reimbursement for visits Monitoring of telehealth use at payor level with a health equity lens

Going forward, the supportive care community can build systematic and collaborative programs of pragmatic research to optimize equitable telehealth clinical models. Such research should continue to shape developments of telehealth in cancer, exploring and testing solutions to address barriers at all levels. Ongoing research programs should evaluate comprehensive cancer care outcomes, patient-reported measures, ease of use, patient engagement, patient preferences, and implementation outcomes with a specific focus on disparity indicators (e.g., reach, adoption, and sustainability). It is also essential that these research programs influence policy across the health care systems. As the peak multi-national association for excellence in cancer supportive care, MASCC will be best placed to develop evidence-based guidance for solutions and implementation strategies to overcome disparity and maximize equity in telehealth for people affected by cancer.

## Data Availability

Not applicable.
